# Mobility between communities can reduce the impact of measles intervention during an outbreak: a mathematical modeling study

**DOI:** 10.3389/fpubh.2026.1794929

**Published:** 2026-05-14

**Authors:** Michael A. Irvine, Kari Harder, Prital Patel, Agatha Jassem, Megan R. Edwards, Elaine Chan, Jia Hu, Jennifer Vines, Trevor Corneil, Tribesty Nguyen, Hind Sbihi

**Affiliations:** 1 BC Centre for Disease Control, Vancouver, BC, Canada; 2 Department of Statistics, University of British Columbia, Vancouver, BC, Canada; 3 Faculty of Health Sciences, Simon Fraser University, Burnaby, BC, Canada; 4 Northern Health, Prince George, BC, Canada; 5 Department of Pathology and Laboratory Medicine, University of British Columbia, Vancouver, BC, Canada; 6 School of Population & Public Health, University of British Columbia, Vancouver, BC, Canada

**Keywords:** active case finding, Bayesian analysis, epidemic model, infectious disease dynamics, measles, population mobility, public health intervention, time-series forecast

## Abstract

Measles resurgence has occurred in a number of settings this year where elimination had been declared. Although under-vaccination of the community is the principal reason for the resurgence additional factors confound this further including community structure and mobility between under-vaccinated communities. We conducted a mathematical modeling study of the measles epidemic in Northern British Columbia, Canada during 2025. We constructed a stochastic SEIR-like model and fitted to two separate regions that experienced an outbreak using an approximate Bayesian computation rejection scheme. The calibrated model of the two regions were then used to construct a series of scenarios reflective of the epidemiological and public health situation including active case finding, post-exposure prophylaxis (PEP), population mobility, and the start of the school semester. Within this setting, inter-region mobility can increase the probability of an outbreak by as much as 50%. There was also a 46% increase of an outbreak if the initial case occurred during the school term. However, a combination of PEP and active case finding reduces this probability across all scenarios. Public health intervention can limit the potential for outbreaks, however the broader structure of the community needs to be considered to determine regions with maximum outbreak potential.

## 1 Introduction

Measles is a highly contagious vaccine preventable disease. Pre-immunization, global numbers of deaths due to measles was an estimated 2.6 million deaths per year (1). With the introduction of the measles vaccine and the following mumps-measles-rubella (MMR) vaccine rates have decreased globally by 57% from 2000 to 2024 (2). This vaccine is highly effective at reducing transmission with an effectiveness of reducing onward transmission by 95% after one dose and 96% after two doses (3). However, recent vaccine rates have waned globally including in developed countries such as North America and Canada. Measles was declared eliminated in 2000 in the US and 1998 in Canada, however Canada has lost its elimination status in 2025 and the US is at-risk of losing its status in 2026 due to on-going community transmission of the virus (4, 5). The recent measles North American outbreak has 1439 reported cases in the US as of epi-week 36, 2025 (6). This resurgence of measles has prompted public health organizations to consider questions that have been dormant for decades, specifically what preventive actions are the most effective at reducing infections and severe outcomes (7).

Given the high effectiveness of the measles vaccine, the majority of cases occur among unvaccinated individuals; in Canada, 89% of recent measles cases have been reported in this group (8). Therefore, the recent resurgence of measles cases is directly attributable to communities with low vaccination rates alongside the broad decline in overall vaccination rates (9, 10). This downward trend in vaccine uptake have been exacerbated by the COVID-19 pandemic, which contributed to increased hesitancy across multiple vaccine products including for measles (11, 12). In the US this has led a decline in kindergarten vaccination rates from 95.2% during the 2019-2020 school year to 92.7% during the 2023–2024 school year (13). Although estimates of the herd immunity threshold for measles vary, its exceptionally high transmissibility necessitates coverage of 95% or higher (14). However, recent serological surveys in Canada indicate that population-level immunity remains at approximately 90%, falling below these theoretical thresholds (15).

Mathematical modeling has been an invaluable tool for public health to understand the dynamics of measles resurgence and the impact of further intervention and vaccination (16). Recently Masters et al. explored an outbreak of measles among a migrant shelter in Chicago between February-April 2024 using a variation of a stochastic modeling approach (17). Importantly, they were able to update their projections of measles cases in real-time passing this information along to public health officials managing the outbreak. Recent mathematical modeling has highlighted the geospatial heterogeneity in vaccination coverage and projected current trends in vaccination could lead to increased probability of endemicity, however increasing uptake of first dose could reduce cases by 15% (16, 18).

Despite prior work on the mathematical modeling of measles transmission, important questions remain on the combined impact of public health interventions, population structure, and mobility on case incidence and hospitalizations during periods of resurgence. In particular there are open questions on how mobility between communities and school-based contacts can reduce the effectiveness of intervention. We present a recent mathematical modeling analysis of a measles outbreak in rural communities in British Columbia (BC), Canada. We developed an age-structured stochastic SEIR model and performed a series of Bayesian fitting to case data of outbreaks in two distinct communities, enabling real-time assessment to support public health decision-making. We validated the model using projections of cases and hospitalizations across the two regions. We then explored scenario analyses of interventions and epidemiological context to support public health decision making including the use of post-exposure prophylaxis (PEP) and active case finding, changes in population mobility, and whether schools are in-session.

## 2 Methods

### 2.1 Model overview

We adapted a measles transmission model suitable for simulating transmission in small to medium sized communities (17). The model incorporates vaccine history, age structure, natural history of infection, and public health intervention including active case finding and further vaccination. At the start of a simulation individuals are either in a susceptible or vaccinated state. If an individual is infected then they moved into an exposed state and then an infectious state where they contribute toward the population force of infection via age-age assortativity rates. After their infection they move into a recovered state where they are assumed to be immune.

Model parametrization was performed using a combination of values from the literature and model fitting (17, 19). Analysis of historical measles cases have shown strong age assortativity, which is derived from a combination of factors related to underlying susceptibility, immunity, and contacts within a given age group (20). We incorporate age assortativity into the model by defining a contact matrix Φ, where the entries of the matrix represent the proportional rate of contact between different age groups and the diagonal entries represent the contact within each age group.

We assumed a constant rate of case ascertainment across all age groups. Although the rate at which measles-like illness is investigated varies by age (21), and clinical reporting differ across age groups, with higher severity observed among young children, older adults, and pregnant people (22), we attributed observed age-specific differences in reported cases primarily to underlying variation in immunity rather than differential ascertainment. This assumption is supported by recent case analyses indicating approximately constant case ascertainment across age groups (23).

The model is structured as a continuous time Markov chain with rates that are dependent on the current model state. More concretely a state of the model *X*(*t*) is a random variable that is drawn from a distribution that is characterized by an initial condition vector and a rate matrix. The population is divided into *m* age groups indexed by *i* from 0 to *m*−1. Individuals start in a susceptible state *S*_*i*_ or a vaccinated state *V*_*i*_ and can then progress to an exposed state *E*_*i*_ at a rate which is dependent on the underlying infectivity β, the size of the population in each age group *N*_*i*_, the number of infected individuals *I*_*i*_, and the contact rate from age group *i* to age group *j*, ϕ_*ij*_. The force of infection term for age group *i* is then given by,


$$\begin{array}{l} \lambda_{i}=\beta \underset{j}{\sum }\phi_{ij}\frac{I_{j}}{N_{j}}. \end{array}$$

This represents the proportional rate of contact age group *i* has with age group *j* ϕ_*ij*_, the probability that the contact is infected *I*_*j*_/*N*_*j*_ and the combined rate of contact and probability of an infection per contact β. Once exposed an individual can then become infected at rate σ, and an infected individual recovers at rate γ into state *R*_*i*_. An infected individual can also be reported as a case with probability *p*. The state *D*_*i*_ represents individuals who have been infected and will be reported at a given time. An individual in state *D*_*i*_ will then become a case *C*_*i*_ at rate δ, which represents the delay in testing and reporting. We also modeled the number of hospitalized individuals *H*_*i*_ by age group *i*. We used literature-derived estimates of the proportion of infections that are hospitalized *h*_*i*_ by age group *i*, and assume negligible rates of primary vaccine failure so that rates are concentrated in non-vaccinated individuals only (Table 1). Hospitalizations are assumed to occur after symptom onset from the *I*_*i*_ compartment and an individual no longer contributes to the force of infection once hospitalized. Considering the relative rates of recovery and hospitalization, the rate of hospitalization by age group *i* is given as $\gamma \frac{h_{i}}{1-h_{i}}$.

**Table 1:** Model parameter values.

Parameter	Value	References
Fixed parameters		
Incubation period (days)	11	(28, 43)
Infectious period (days)	8	(19, 44)
Ig PEP effectiveness (%)	100	(34)
Fitted parameters		
Infectivity (days^−1^) β	*N*_[0, ∞)_(μ = 0.17, σ = 0.03)	Prior constructed so 95% of mass falls within range 12-18 (45)
Case ascertainment	*N*_[0, 1]_(μ = 0.5, σ = 0.2) (A) *N*_[0, 1]_(μ = 0.8, σ = 0.05) (B)	
Immunization parameters		
< 1	0.0 (*A*) 0.08 (*B*)	Assumed maternal antibody derived immunity (46)
1–5	0.05 (*A*) 0.6 (*B*)	
5–12	0.05 (*A*) 0.77 (*B*)	
13–18	0.05 (*A*) 0.78 (*B*)	
19–54	*N*_[0, 1]_(μ = 0.874, σ = 0.1)	Based on national study of immunization, with mean rate of 87.4% (47)
55+	100	Assumed fully immunized due to prior infection
Population sizes		
< 1	7 (*A*) 313 (*B*)	
1–5	15 (*A*) 598 (*B*)	
5–12	29 (*A*) 1,445 (*B*)	
13–18	24 (*A*) 1,508 (*B*)	
19–54	157 (*A*) 9,787 (*B*)	
55+	69 (*A*) 6,349 (*B*)	
Hospitalization rates		(48)
< 1	0.133	
1–5	0.105	
5–12	0.04	
13–18	0.055	
19–54	0.144	
55+	N/A	Immunization rate assumed to be 100%, so not applicable

Priors are given using their corresponding statistical distributions and region-specific parameters are highlighted with the region labels *A* and *B*. Unless otherwise indicating other parameters are the same across regions.

We also considered how PEP through administering immunoglobulin G (IgG) impacts onward transmission. We assumed that PEP is administered to an individual who is un-immunized (susceptible in the model) and that this occurs following a potential exposure. The individual is then no longer susceptible for the duration of the outbreak. We set the effectiveness of PEP at 100% and vary the time from being exposed to receiving PEP while in the exposed state (24). Given that the time in the exposed state is exponentially distributed (*T*_*E*_) and the time to PEP use is also exponentially distributed with rate ι, (*T*_*PEP*_), the probability that PEP is administered before leaving the exposed state is $P\left(T_{PEP}<T_{E}\right)=\frac{\iota }{\iota +\sigma }$. We can therefore parameterize the rate of PEP as the probability that it is administered while an individual is exposed.

**Figure 1:**
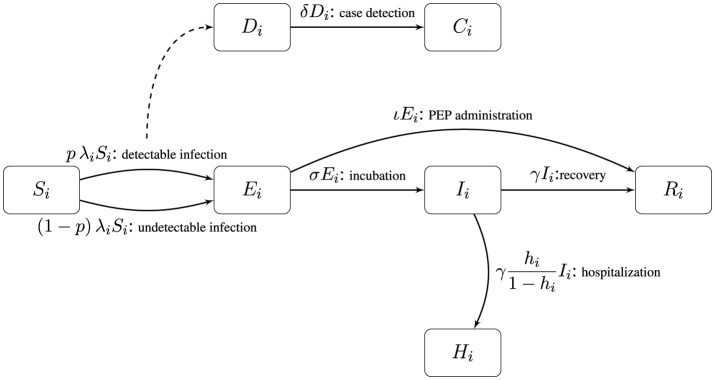
Model flow diagram. Rates are provided along with the name of each transition. Dashed lines denote a transition that does not deplete the parent state. Individuals in group *i* start as susceptible (*S*_*i*_) unless vaccinated (state not shown) and then progress to exposed (*E*_*i*_) and then either receive PEP and transition to recovered (*R*_*i*_) or become infectious (*I*_*i*_). From the infectious state an individual either recovers or transitions to being hospitalized (*H*_*i*_).

The complete set of model transitions and their rates are as follows with a model diagram provided in Figure 1,


$$\begin{array}{l} \begin{array}{lll} \left(S_{i},E_{i},D_{i}\right)\to \left(S_{i}-1,E_{i}+1,D_{i}+1\right) & \text{at rate }p\lambda_{i}S_{i} & \text{(detectable infection)},\\ \left(S_{i},E_{i},D_{i}\right)\to \left(S_{i}-1,E_{i}+1,D_{i}\right) & \text{at rate }\left(1-p\right)\lambda_{i}S_{i} & \text{(undetectable infection)},\\ \;\left(E_{i},I_{i}\right)\to \left(E_{i}-1,I_{i}+1\right) & \text{at rate }\sigma E_{i} & \text{(incubation)},\\ \;\left(I_{i},R_{i}\right)\to \left(I_{i}-1,R_{i}+1\right) & \text{at rate }\gamma I_{i} & \text{(recovery)},\\ \;\left(D_{i},C_{i}\right)\to \left(D_{i}-1,C_{i}+1\right) & \text{at rate }\delta D_{i} & \text{(case detection)},\\ \;\left(H_{i},I_{i}\right)\to \left(H_{i}+1,I_{i}-1\right) & \text{at rate }\gamma \frac{h_{i}}{1-h_{i}} & \text{(hospitalization)}\\ \;\left(E_{i},R_{i}\right)\to \left(E_{i}-1,R_{i}+1\right) & \text{at rate }\iota E_{i} & \text{(PEP administration)}. \end{array} \end{array}$$

This model assumes that individuals that are vaccinated cannot become infected and do not contribute toward onward transmission. A recent meta-analysis indicates that the rate of viral shedding among secondary vaccine failure cases is low and unlikely to lead to new infections (25).

The reproduction number in the absence of intervention and in a completely susceptible population (*R*_0_) can be calculated using the next generation matrix approach (26, 27). This procedure follows by linearizing the system around the disease-free equilibrium and then defining the new infections matrix *F* and the rate of transitions between infected compartments *V*. Since under no intervention, all individuals who are exposed necessarily become infectious, *F* can be simplified to include only the *I*_*i*_ components and can be written as,


$$\begin{array}{l} F_{ij}=\beta \phi_{ij}\frac{N_{i}}{N_{j}}, \end{array}$$

which represents the rate of new infections in group *i* from infectees in group *j*. Since no further transitions occur once an individual is infectious to when they recover, the rate of transitions between infectious compartments *V* can be written as *V* = γ*I*, where *I* is the identity matrix. $V_{ij}^{-1}$ therefore represents the expected duration of an individual in group *i* starting in group *j*, which is *V*^−1^ = γ^−1^*I*. The next generation matrix is defined as *K* = *FV*^−1^ and each (*i, j*) entry represents the expected number of new infections generated in group *i* by an infection in group *j*. The spectral radius of the next-generation matrix ρ(*K*) defines the basic reproduction number *R*_0_ (27), which given the properties of the spectral radius is,


$$\begin{array}{l} R_{0}=\frac{\beta }{\gamma }\rho \left(T\right). \end{array}$$

Where *T* is the contact matrix transformed by the ratio of population sizes (14),


$$\begin{array}{l} T_{ij}=\phi_{ij}\frac{N_{i}}{N_{j}}. \end{array}$$

We used this relationship to parameterize β directly from literature-based estimates of *R*_0_ for measles.

### 2.2 Multi-region analysis

We considered two regions with two distinct population sizes and contact age structure. We focused on two regions as the 2025 Northern BC outbreak was predominantly concentrated there. Although extending the model to additional regions could produce more complex or granular scenarios, restricting the analysis to the most affected areas allowed a clearer assessment of the effects of inter-regional mobility on measles transmission in the absence of comprehensive mobility data. Given the study context, we were limited to incorporating region and age-assortative mixing only; however, future work should explore more fine-grained contact network structures across multiple communities to better capture heterogeneity in transmission pathways. We first labeled the regions as *A* and *B* with an associated contact matrix $\Phi^{A}={\left(\phi_{ij}\right)}_{j\in \left\{0,\ldots ,m-1\right\}}^{i\in \left\{0,\ldots ,m-1\right\}}$ in region *A* and an equivalent contact matrix Φ^*B*^ in region *B*. In addition, we considered the rate of contact between an individual in age-group *i* and region *A* with an individual in age-group *j* and region *B*. We further defined the overall contact matrix using the contact matrix Φ^*AB*^ and Φ^*BA*^ for contact from region *A* to region *B* and from region *B* to region *A* respectively.

We modified the force of infection term for an individual in age-group *i* and region *A* as,


$$\begin{array}{l} \lambda_{i}^{A}=\beta \left(\underset{j}{\sum }\phi_{ij}^{A}\frac{S_{j}^{A}}{N_{j}^{A}}+\underset{j}{\sum }\phi_{ij}^{AB}\frac{S_{j}^{B}}{N_{j}^{B}}\right), \end{array}$$

and similarly for region *B*,


$$\begin{array}{l} \lambda_{i}^{B}=\beta \left(\underset{j}{\sum }\phi_{ij}^{B}\frac{S_{j}^{B}}{N_{j}^{B}}+\underset{j}{\sum }\phi_{ij}^{BA}\frac{S_{j}^{A}}{N_{j}^{A}}\right). \end{array}$$

We simplified the overall contact matrix by assuming proportionally constant contact between communities i.e.,

$\phi_{ij}^{AB}=\rho_{AB}\phi_{ij}^{B}$ and $\phi_{ij}^{BA}=\rho_{BA}\phi_{ij}^{A}$.

We then re-index such that *i* represents both age-group and region. The contact matrix under this re-indexing has the following structure,


$$\begin{array}{l} \text{Φ}=\left(\begin{array}{cc} \left[\begin{array}{c} \phi_{ij}^{A} \end{array}\right] & \left[\begin{array}{c} \rho_{AB}\phi_{ij}^{B} \end{array}\right]\\ \left[\begin{array}{c} \rho_{BA}\phi_{ij}^{A} \end{array}\right] & \left[\begin{array}{c} \phi_{ij}^{B} \end{array}\right] \end{array}\right) \end{array}$$

We further simplified by assuming constant mobility between regions, ρ_*AB*_ = ρ_*BA*_ = ρ.

We considered two public health interventions during an outbreak: active case finding and PEP. Active case finding was assumed to reduce the effective duration of infectiousness through earlier diagnosis and isolation of cases. We considered a range of how active case finding reduces the infectious duration between 8 days and 4 days, which would coincide with the earliest time of symptom onset after infection (28).

### 2.3 Data

We extracted reporting line-lists for the two regions where a sustained outbreak of measles cases occurred in the Northern Health region in British Columbia in 2025. Given the sensitivity of measles immunization status in these communities we preserved the anonymity of these regions and label them as *A* and *B*. We extracted all PCR-confirmed reportable cases for region *A* from 2025-05-31 to 2025-08-13 and region *B* from 2025-06-19 to 2025-11-02 to encompass the main period of outbreak in both regions. Cases were then aggregated by date of rash onset and into age categories < 1, 1-4, 5-11, 12-17, 18-54, and 55+. These age categories were used to account for the heterogeneity in contact patterns, vaccine history, epidemiology, and morbidity associated with measles and aligns with age categories from other public health agencies (8). Cases were then further filtered on whether they had an associated hospitalization to produce the hospitalization incidence in region *A* and *B*.

### 2.4 Model fitting

We assumed that the population size of each community was fixed and the population age was distributed based on the estimates of the closest health region for region *A* and *B* (29). The contact matrix was defined using POLYMOD data adjusted for the local population distribution (Supplementary Figure 4) (30, 31). Population immunity was derived from the Provincial Immunization Registry (PIR) for pediatric age groups and from the Canadian national survey on immunization for adults aged 19–54. Given the greater uncertainty around the immunization rate, this value was not fixed and given an associated prior. We based infants < 1 immunization rates on the assumed maternal antibody derived immunity rate of immunized females of child-bearing age. Adults 55+ were assumed to be immunized due to prior infection. A summary of assumed immunity rates is shown in Table 1. Cases by rash onset date were fit for region *A* up to 2025-06-22 and for region *B* up to 2025-08-13. This was reflective of the real-time model fitting and projection analysis that was provided to public health officials during the outbreak in each region. Model fitting was conducted using a simple approximate Bayesian computation (ABC) rejection scheme based on the summary statistics of cumulative cases by rash onset date and age group (32). This scheme was chosen as it allowed for faster model fitting and projections to be produced compared with more accurate, but computationally slower Bayesian sampling schemes (33). We selected 10,000 initial samples of the prior for each region and set a tolerance of 10% to produce 1,000 approximate samples of the posterior.

### 2.5 Model scenarios

We explored a number of counterfactual scenarios to understand the impact of available public health measures and contextual factors, including differences in mobility between communities and the timing of school semesters. In order to reduce within-scenario variance, fixed values were used for case ascertainment and immunization rates, whilst infectivity was varied across the model prior. Fixed immunization rates for the 18–54 age group were set using the posterior estimates specific to each region. The scenarios were combined to understand the total impact of public health interventions in the specific settings of the school session status and region. Each scenario was simulated for 90 days across 1,000 realizations and each started with an initial condition where a single individual was infected in the 12–17 age group in each region.

#### 2.5.1 Active case finding

Three scenarios were considered for active case finding and its impact on the effective duration of the infectious period. These were whether active case finding reduced the duration by 0%, 25% or 50% equivalent to an expected infectious period of 8, 6, and 4 days respectively.

#### 2.5.2 Post-exposure prophylaxis

Estimates of the effectiveness of Ig PEP vary from 83.4 to 100% (34). For the purpose of the study scenarios we assume a 100% effectiveness of PEP and based scenarios around the proportion of PEP that is effectively administered when an individual is exposed, but before becoming infectious. These were whether PEP is used effectively by 0%, 25%, or 50% of exposed individuals.

#### 2.5.3 School session

To assess the impact of school session status, we extracted the age-dependent contact matrix for all contacts that occur outside of a school setting (non-school scenario) and all contacts including within a school setting (school scenario). To enable a consistent comparison between these scenarios under a fixed transmission framework, we calculated the infectivity β required to produce an equivalent *R*_0_ range in the non-school scenario and used the same infectivity rate for the school scenarios. β was derived using the described next generation matrix approach drawing from the prior distribution of the basic reproduction number (*R*_0_).

#### 2.5.4 Community mobility

The community mobility parameter ρ was used to develop four scenarios of inter-region contact based on the scaled rate of contact within and between contacts. These were where: no contact occurred between communities, 1 in 100, 1 in 20 or 1 in 10 contacts were between regions *A* and *B*. The relationship between proportion of contacts between region (*p*) and mobility rate (ρ) is $\rho =\frac{p}{1-p}$ by definition.

## 3 Results

**Figure 2:**
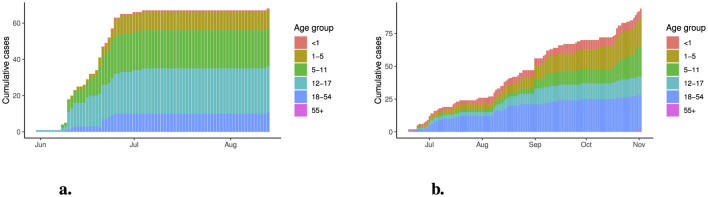
Cumulative number of reported cases by rash onset date for **(a)** region *A* and **(b)** region *B*.

The distribution of cases by age group and the duration of the outbreak both varied by region *A* and *B* (Figure 2). In region *A*, which was the smaller of the two regions (*n* = 300) the outbreak occurred over a five-week period with the majority of cases in the 1-17 age groups. Region *B* (*n* = 10, 000) saw a higher proportion of cases in the < 1 and 18–54 age group with a sporadic increase in cases over a 16 week period.

**Figure 3:**
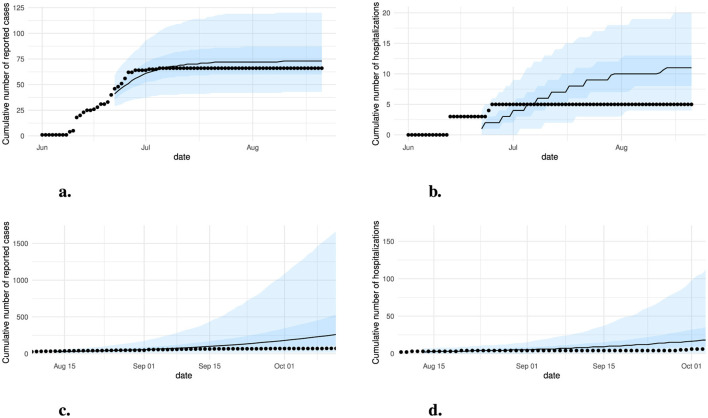
Validation plots for region *A* and *B*. Reported cases and hospitalizations are provided as black points and model projections based on posterior estimates are provided as medians (line), 50% credible interval (darker shading) and 95% credible interval (lighter shading). **(a)** cases for region *A*, **(b)** hospitalizations for region *A*, **(c)** cases for region *B*, and **(d)** hospitalizations for region *B*.

The model was fit independently to region *A* and region *B*. The infectivity (β) and the case ascertainment (*c*) posterior distributions were not updated substantially from their priors (Supplementary Figure 1). The 18–54 immunization rate however showed a greater difference between the prior and posterior for region *B* with posterior estimate 83% (95% CrI: 71–91) and a relatively similar rate for region *A* with posterior estimate 76% (95% CrI: 55–96). These differences can be explained by the number of cases experienced in each region which contributes as evidence toward the posterior. The model was well calibrated in both regions with posteriors overlapping with cumulative cases by age group (Supplementary Figure 2). Within the model validation cases closely matched the projection in region *A*, but were lower than the expected cases in region *B* (Figure 3). Although in region *B*, the 95% uncertainty interval did overlap with the number of cases, this was mainly driven by the large uncertainty in whether a large proportion of the population would become infected during the course of the outbreak. The cumulative number of hospitalizations did overlap with the projections, however the expected number of hospitalizations was higher in both regions.

### 3.1 Probability of outbreak by scenario

**Figure 4:**
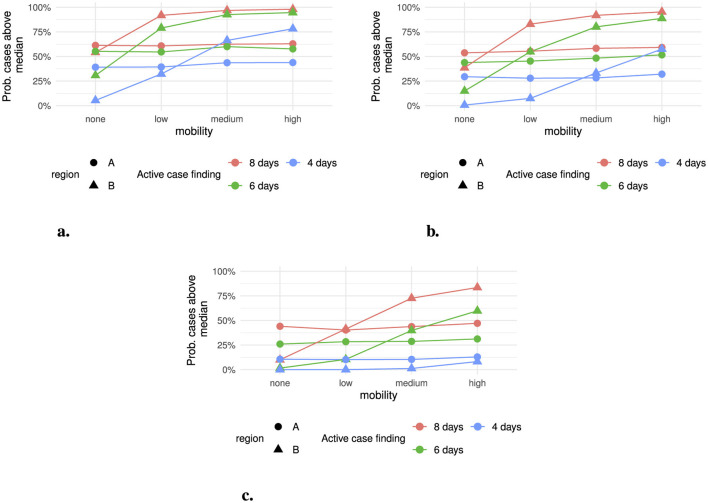
Probability of outbreak exceeding the median number of cases within a region across all scenarios and simulations. **(a)** PEP 0% effective, **(b)** PEP 25% effective, and **(c)** PEP 50% effective.

Within the scenario analysis, mobility between regions increased the probability of an outbreak despite intervention (Figure 4). Within scenarios where no PEP was distributed, the probability of an outbreak varied between 5-54% in region *B* for different levels of active case finding where there is no mobility between regions. Region *A* had a similar probability of an outbreak, between 40-63%, across active case finding and mobility scenarios. With increasing mobility between regions, the probability of an outbreak in region *B* increased to 78% for the highest level of active case finding and 98% where there was no active case finding. The use of PEP alone reduces the probability of an outbreak in region *A* to 47-59% across mobility scenarios (Figures 4b and c). In region *B*, the use of PEP reduces the probability of an outbreak in all mobility scenarios, however this reduction is limited in the high mobility scenario.

### 3.2 Distribution of cases & hospitalizations by scenario

**Figure 5:**
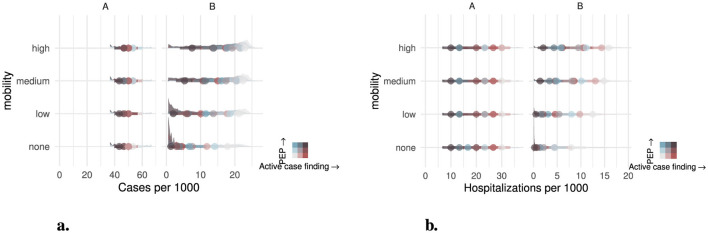
Distribution of total outcomes by region (left and right), mobility (row), Active case finding, and PEP use (colour). Distribution of total outcomes by scenario are shown as distribution plots, with median and inter-quartile range shown as points and bars respectively. **(a)** Cases per 1,000 population and **(b)** Hospitalizations per 1,000. To enhance distinction between scenarios, simulations that didn't result in an outbreak were suppressed.

Mobility also reduced the impact of public health intervention on the final distribution of cases over an outbreak (Figure 5a). Although region *A* is broadly unaffected by the impact of mobility due to its low vaccination rate, it greatly impacts scenarios in region *B*. Where there is no contact between regions, the highest number of cases occurs for the 0% PEP and 8 day active case finding scenario with 18 cases per 1,000. Greater PEP or increased active case finding reduced the number of cases to 6.7 and 4.5 per 1,000 respectively. Cases were further reduced when PEP and active case finding are combined to 1.3 cases per 1,000. However, even with low mobility, the rate of cases increased to 21 per 1,000 without intervention. Active case finding or PEP alone only reduced the total cases to 10 and 12 per 1,000 respectively. The combined interventions were still able to reduce cases to a similar rate where there was no mobility at 2.0 cases per 1,000. For high mobility however, the total cases increased to 23 per 1,000 with no intervention, reducing only to 17 and 18 per 1,000 for active case finding and PEP respectively. Even with the highest level of active case finding and PEP, the total cases were only reduced to 7.5 per 1,000. A similar pattern to cases was also found for hospitalizations in the final distribution by scenario (Figure 5b). Mobility did not impact region *A* where the rate varied between 30.0 to 10.0 per 1,000 for different levels of intervention. For region *B* with no intervention the rate of hospitalizations varied from 7.95 to 15.85 per 1,000 from the lowest to highest rate of mobility. With the highest rate of intervention, the number of cases varied from 0.20 to 2.05 per 1,000 from the lowest to the highest rate of mobility.

### 3.3 Distribution of cases by age group

**Figure 6:**
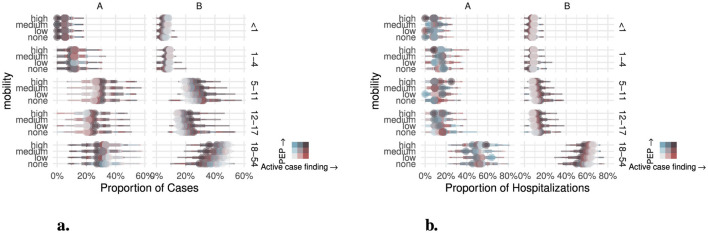
Proportion of outcomes by age-group, mobility and intervention scenarios for **(a)** cases **(b)** hospitalizations. To enhance distinction between scenarios, simulations that did not result in an outbreak were suppressed.

The proportion of cases by age group also shifted proportionally by intervention and mobility (Figure 6a). In region *A*, the proportion of cases by age group was mainly affected by intervention, but remained stable under change in mobility. For < 1 and 1–4 age groups, the proportion was 5–6% and 11–13% under PEP respectively. There was more variation in the 5–11 and 12–17 age groups, at 25% and 20% at no intervention to 31% and 25% with combined PEP and active case finding at the highest rates. In the adults (18–54), increasing intervention reduced the proportional number of cases from 35% to 27%. Note that although the proportion of cases varied between age groups, the overall number of cases was lower with increasing intervention. The proportion of cases among age groups was more impacted by mobility in region *B*. For no intervention, the proportion of cases in 5–11 and 12–17 decreased as mobility increased from 22% and 16% to 19% and 14% respectively. In the 18–54 age group, the proportion increased from 45-50%. Under the combined PEP and active case finding scenarios, the proportion in 5–11 decreased from 31% to 28% as mobility increased, with the proportion in 12–17 age group decreasing from 26% to 23%, and the proportion in 18–54 increasing from 29% to 36%.

The proportion of hospitalizations by age group in region *A* had less consistent variation by scenario with < 1 from 0–9%, 1–4 from 12–18%, 5–11 from 8–12%, 12–17 from 9–17% and 18–54 from 45–55% (Figure 6b). In region *B* intervention and mobility increased the proportion of hospitalizations in children (12–17: 8–15%) and decreased the proportion in adults (18–54: 66–50%).

### 3.4 Impact of school session status

**Figure 7:**
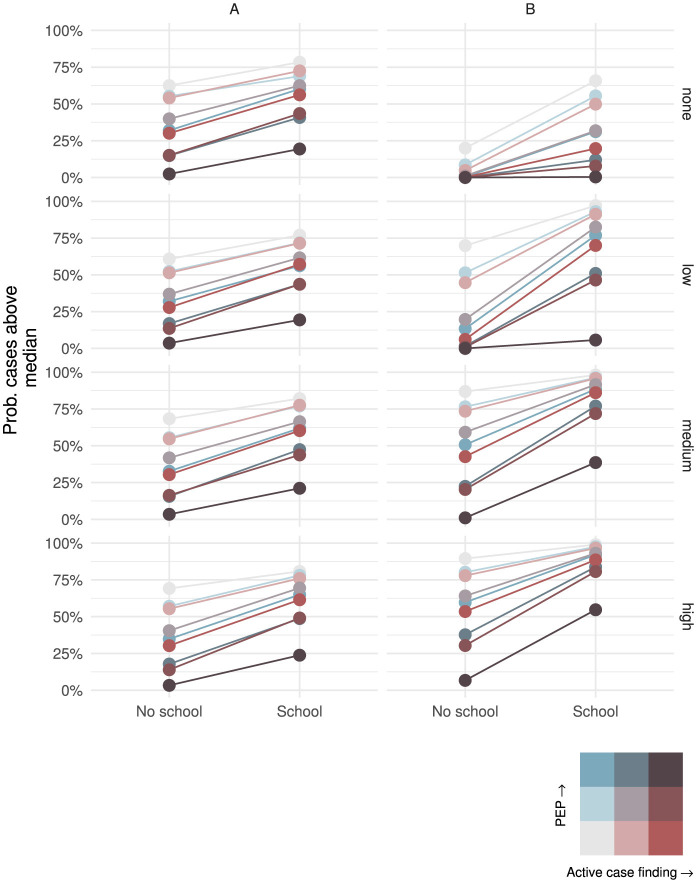
Probability of outbreak exceeding the median number of cases within a region (columns) by school contact (x-axis), mobility (rows) and intervention (colour).

No school contact during an outbreak lowered the probability of an outbreak in both regions, however this was moderated by mobility and the strength of intervention (Figure 7). With no mobility between regions, in region *A* without intervention, the probability of outbreak increased from 62% to 78% by session status. With combined high PEP and active case finding, school contact increased the probability from 2% to 19%. Region *B* had a greater change in the probability of an outbreak with school contacts from 20% to 66% where there were no interventions. However, with combined high PEP and active case finding, the probability of outbreak is unaffected by school session status (0% in both scenarios). Increased mobility did not affect the difference in probability of an outbreak in region *A*, but did lead to an increased probability across interventions in region *B* with the exception of the highest intervention scenario. For example, with medium mobility and no intervention the probability of an outbreak increased from 87% without school-based contact to 98% with school-based contact. Without PEP and with high active case finding, the increase was from 43% to 86%, and for combined PEP and active case finding at the highest rates, the probability increased from 1% to 39%.

The distribution of total cases was similarly affected by including school contacts increasing from 3.0 to 18 cases per 1,000 in region *B* without any mobility and intervention; from 11 to 21 cases per 1,000 with low mobility; and 21 to 23 cases per 1,000 in the high mobility scenario (See Supplementary Figure 3).

## 4 Discussion

We examined a calibrated and validated model of measles resurgence in distinct community settings, focusing on the roles of interventions and community contact patterns in shaping outbreak probability, as well as the resulting distributions of cases and hospitalizations. We found that mobility between under-vaccinated communities can reduce the impact of public health intervention in reducing an outbreak by 8.2% to 64% depending on the level of intervention. The final distribution of cases is similarly affected with mobility increasing the number of cases between 7.5 to 12.8 per 1,000 population. Whether an outbreak occurs when schools are in session can similarly reduce the impact of intervention, increasing the probability of an outbreak by 17%. We found that although public health intervention has the potential to greatly decrease measles infections and subsequent hospitalizations, important community factors need to be considered in terms of their potential impact.

We adopted a stochastic simulation approach, which is suitable for modeling of a measles outbreak within a finite population and has been applied in recent analyses (17). Our approach used a continuous-time Markov chain, which captures greater realism such as early extinction of an outbreak, and more realistic trajectories of measles cases compared with other stochastic approaches (35, 36).

We found large differences in the attack rate and duration of outbreak in measles within two communities in British Columbia, Canada. These differences can be explained by pediatric vaccination rates as well as the estimated difference in the rate of vaccination in the adult population between the two communities. We found that the overall age-specific distribution of cases can be broadly explained by similar infection rate and case ascertainment across age groups, combined with heterogeneity in immunization coverage. Although the model projection for region *A* matched with the total number of cases, the projection overestimated both cases and subsequent hospitalizations in region *B*. Several factors could explain this discrepancy. Region *A* is smaller than region *B* (population size approximately 300 vs. 10,000) making the model assumptions of proportional mixing within age-groups more well-founded. In contrast, cases in region *B* were more clustered in time with periods of no new cases during the course of the outbreak. This pattern potentially indicates that smaller sub-communities within region *B* experienced a succession of localized outbreaks that would not be easily captured by the model contact structure. Nevertheless, this highlights the large amount of uncertainty present in the forecasts of measles outbreaks in communities that are close to a critical threshold for vaccination (37).

Spatial dynamics have played an important role in measles transmission during the pre-vaccination era, with large urban centers driving infections in surrounding smaller communities (38). However, in the post-vaccine era, measles dynamics are less well characterized by spatial spread. Our study highlights that in the post-vaccine era spatial dynamics can still potentially play a role where communities with low vaccine rates are more proximal to one another. The specific density of surrounding communities and their mobility will in general play a role in the dynamics of measles (39). Nevertheless, as noted by Lau et al. the pattern of which communities will generate new outbreaks in other communities is not generally predictable, and the community-level vaccination rate remains the strongest predictor of an outbreak (38).

There are several important limitations of our study to consider. First, we considered public health interventions at a constant rate throughout the outbreak. In practice, delays between initial infection, case detection, reporting and the initiation of public health action and control measures are inevitable and would likely attenuate the overall impact. However, because our analysis considered a broad range of intervention scenarios, this reduction is expected to fall within the uncertainty bounds of our results. Second, contact structure in the model was limited to age-based assortative mixing and contact between two regions. This simplification neglects additional layers of assortative mixing that are likely to exist, particularly in large regions where transmission may be structured by sub-communities. For example, sub-communities formed by strong social networks of shared heritage, tradition, and ways of life are likely important factors in driving transmission. Given our study context, we could only consider age-assortative mixing, but future studies should consider these more fine-grained contact network structures across multiple communities (40). Our study focused on rural/remote settings where the outbreak was observed and its findings may not necessarily translate to urban settings where there is greater population density and differences in community structure that may affect measles transmission dynamics (41). Finally, we did not explicitly capture the impact of isolation following a laboratory-confirmed positive diagnosis. However, this is expected to be included within an overall effective duration of the infectious period, which was captured with our analysis. Adherence to isolation recommendations following diagnosis remains uncertain and is likely context dependent, with potentially lower compliance in rural or resource-limited settings where structural barriers such as access to childcare may constrain individuals' ability to isolate (42).

With ongoing fluctuations in the uptake of measles vaccine globally, there is an increased probability of measles outbreaks in jurisdictions where it had previously been eliminated. Responding public health agencies may need to assess future outbreak potential in under-immunized communities by also considering immunization rates in surrounding areas. As countries seek to regain measles elimination status, understanding the geospatial population risks will be critical in prioritizing public health preventive actions and informing outbreak response plans.

## Data Availability

The original contributions presented in the study are included in the article/Supplementary material, further inquiries can be directed to the corresponding author.
