# Supplementary material for: Mobility between communities can reduce the impact of measles intervention during an outbreak: a mathematical modeling study
Source: Front Public Health. 2026 May 14;14:1794929. doi: 10.3389/fpubh.2026.1794929 (PMC13216455; doi:10.3389/fpubh.2026.1794929)
Supplement: Supplementary file 1 [file Data_Sheet_1.pdf]

Supplementary Material

MODEL EQUATIONS

The model states are indexed by i, which represents both region and age. The deterministic limit of the continuous-time Markov chain model described in the main text can be written as the following set of ordinary differential equations,

$$
\begin{aligned}
\frac{\mathrm{d}S_i}{\mathrm{d}t} &= -\beta S_i \sum_j \phi_{ij} \frac{I_j}{N_j}, \\
\frac{\mathrm{d}E_i}{\mathrm{d}t} &= \beta S_i \sum_j \phi_{ij} \frac{I_j}{N_j} - \sigma E_i - \iota E_i, \\
\frac{\mathrm{d}I_i}{\mathrm{d}t} &= \sigma E_i - \gamma \frac{h_i}{1-h_i} I_i - \gamma I_i, \\
\frac{\mathrm{d}R_i}{\mathrm{d}t} &= \gamma I_i + \iota E_i, \\
\frac{\mathrm{d}H_i}{\mathrm{d}t} &= \gamma \frac{h_i}{1-h_i} I_i, \\
\frac{\mathrm{d}D_i}{\mathrm{d}t} &= p\beta S_i \sum_j \phi_{ij} \frac{I_j}{N_j} - \delta D_i, \\
\frac{\mathrm{d}C_i}{\mathrm{d}t} &= \delta D_i.
\end{aligned}
$$

As described in the main text the states are susceptible (S_i), exposed (E_i), infected (I_i), recovered (R_i), cumulative hospitalizations (H_i), detectable infection (D_i), and cumulative cases (C_i). β is the infectivity, ϕ_{ij} is the rate of contact to age-region i from j. N_j is the population size of age-region j, σ is the incubation rate, ι is the PEP administration rate, h_i is the proportion of infections that are hospitalized for region-age i, p is the proportion of infections that are detected as cases, and δ is the rate of delay between onset of the infectious period and being identified as a case, conditioned on the infection being identifiable.

SUPPLEMENTARY TABLES AND FIGURES

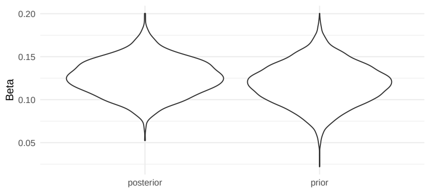

Figure 1a.

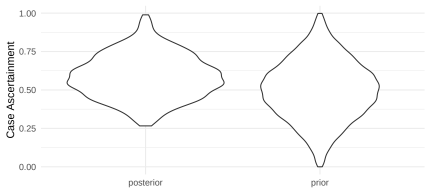

Figure 1b.

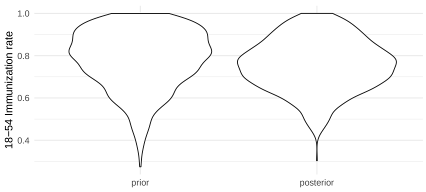

Figure 1c.

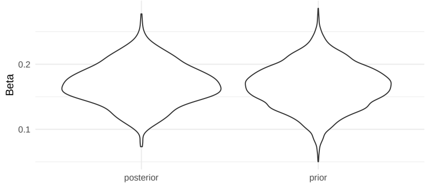

Figure 1d.

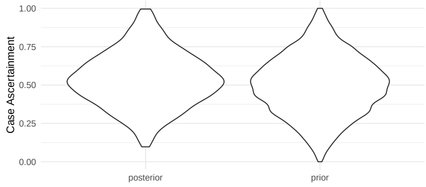

Figure 1e.

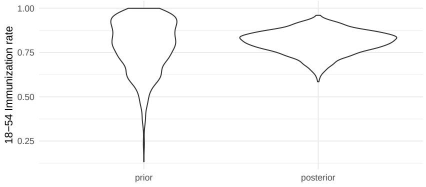

Figure 1f.

Figure 1. Marginal prior and posterior parameters for region A (**a, b, & c**) and region B (**d, e, & f**)

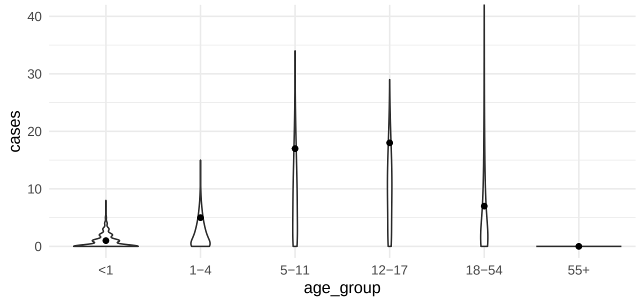

Figure 3a.

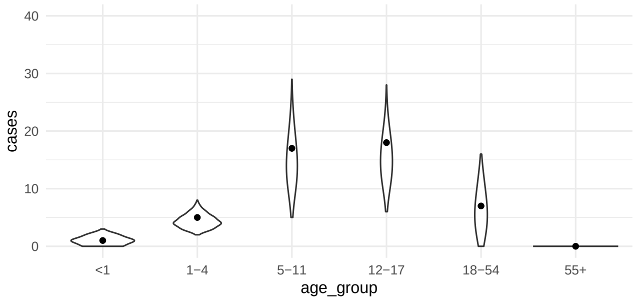

Figure 3b.

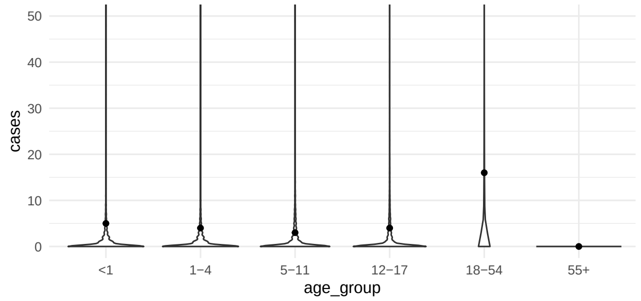

Figure 3c.

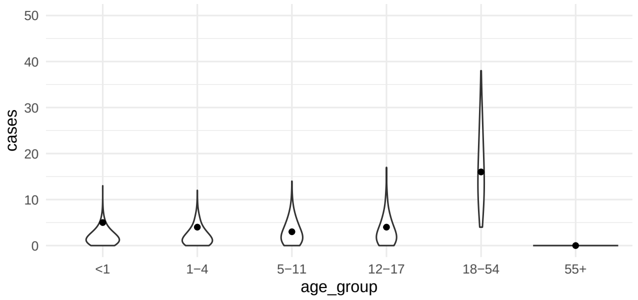

Figure 3d.

Figure 3. Calibration plots of the cumulative number of cases observed (as points) compared to the prior predictive distribution (**a & c**) and posterior predictive distribution (**a & d**) shown as violin distributions for region A (**a & b**) and region B (**c & d**).

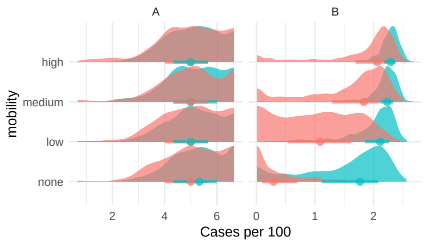

Figure 3a.

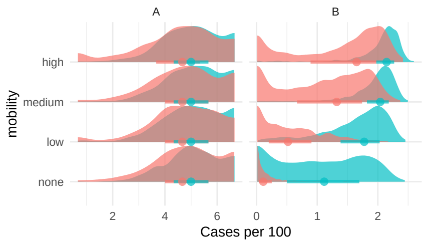

Figure 3b.

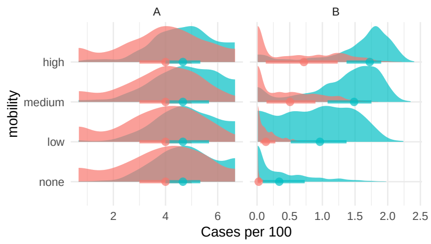

Figure 3c.

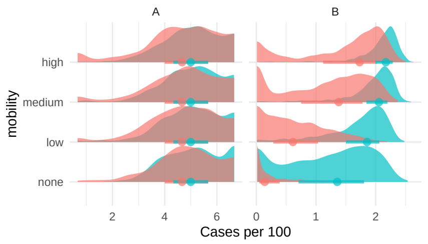

Figure 3d.

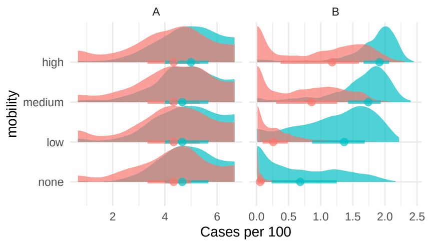

Figure 3e.

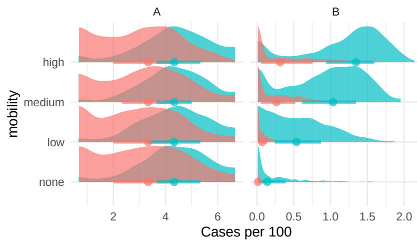

Figure 3f.

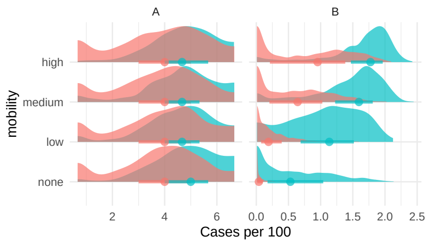

Figure 3g.

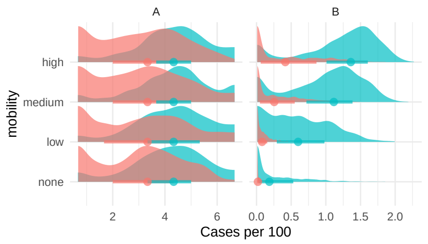

Figure 3h.

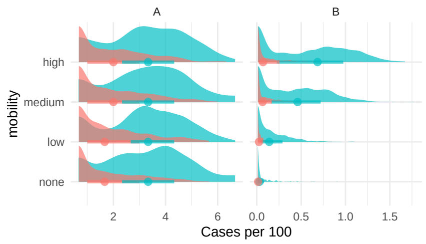

Figure 3i.

Figure 3. Final distribution of cases including school-based contacts (shown in blue) or excluding school-based contacts (shown in red) by region A and B. Interventions are divided into rows and columns with the first row where 0% PEP is distributed (**a, b & c**), the second row where 25% PEP is distributed (**d, e & f**), and the third row where 50 PEP is distributed (**g, h & i**). The first column is where active case finding results in an expected 8 day infectious period (**a, d & g**), the second column is a 6 day infectious period (**b, e & h**) and the third column is a 4 day infectious period (**c, f & i**)

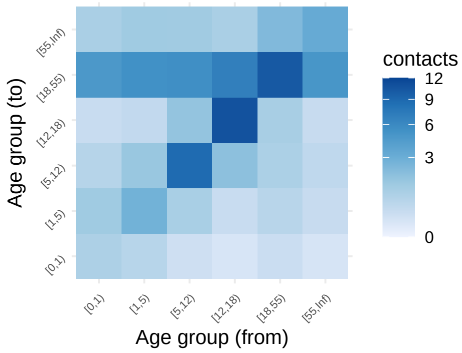

Figure 4a.

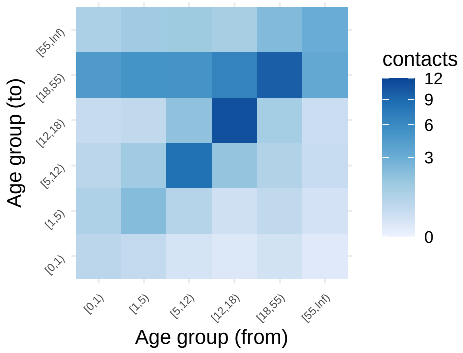

Figure 4b.

Figure 4. Synthetic contact matrices used for **(a)** region A and **(b)** region B.
